# Ebola in West Africa

**DOI:** 10.3889/oamjms.2015.013

**Published:** 2015-02-09

**Authors:** Lul Raka, Monica Guardo

**Affiliations:** 1 *National Institute of Public Health of Kosovo and Medical School, University of Prishtina, Prishtina, Kosovo*; 2 *Advisor, IHR, Epidemic Alert and Response, and Water Borne Diseases Unit(IR), Communicable Diseases and Health Analysis Department (CHA), Pan American health Organization/World Health Organization-PAHO/WHO, Panama City, Panama*

**Keywords:** ebola, outbreak, West Africa, World Health Organization

## Abstract

Ebola viral disease (EVD) is a severe and life-threatening disease. The current Ebola outbreak in West Africa entered its second year and is unprecedented because it is the largest one in history, involved urban centers and affected a large number of health care workers. It quickly escalated from medical into a humanitarian, social, economic, and security crisis. The primary pillars to prevent EVD are: early diagnosis, isolation of patients, contact tracing and monitoring, safe burials, infection prevention and control and social mobilization. The implementation of all these components was challenged in the field. Key lessons from this Ebola outbreak are that countries with weak health care systems can’t withstand the major outbreaks; preparedness to treat the first confirmed cases is a national emergency; all control measures must be coordinated together and community engagement is the great factor to combat this disease.

Ebola viral disease (EVD) is a severe and life-threatening disease affecting humans and nonhuman primates. Ebola first appeared in 1976 in two simulateous outbreaks in Democratic Republic of Congo and Sudan [1]. Ebola virus is an RNA virus of the Filoviridae family. The origin of the virus is unknown, but fruit bats of Pteripodidae family are considered to be reservoirs of the virus. EVD spreads through direct contact with body fluids (stool, vomit, blood, urine, saliva, semen, breast milk) of a sick person with EVD [2]. The disease can also be transmitted through direct contact with the deceased person’s body during funeral or burial preparation or ceremonies. Infection occurs through broken skin or mucous membranes of the mouth, nose and eyes. Ebola can be contracted also by contact with surfaces or equipment contaminated by body fluids of an infected person. Ebola is not spread by people who don’t have signs and symptoms of disease. A patient is not infective during the incubation period. Ebola is not an airborne disease [3].

The epidemic of Ebola in West Africa is the 25^th^ outbreak, which started in December 2013 in Guinea. The World Health Organization (WHO) was notified of an outbreak in March 2014. In August the WHO declared it an epidemic and a “public health emergency of international concern”. This Ebola epidemic is different from all others because it is the first one to appear in West Africa, is the largest and longest and involves urban centers including capital cities. Until the end of January 2015 there have been 22,495 reported cases of EVD, causing around 8981 deaths [4]. The majority of patients were 15 to 44 years of age (57.7%) and the case fatality rate was 70.8%. The most affected countries are Liberia, Sierra Leone and Guinea. This outbreak was also characterized with aggressive transmission among health care workers (HCW). A total of 816 HCW were infected causing 488 deaths. The clinical course of infection and virus dissemination are similar to that reported in previous outbreaks of EVD. The estimated basic reproduction numbers (*R0*) is 1.8, meaning that one sick person on average infects 1.8 people in his neighbourhood [5].

The incubation period for Ebola is 2 to 21 days. During this outbreak the majority of patients had incubation periods of 11 days. Symptoms and signs of disease are fever, intense weakness, muscle pain, headache and sore throat followed by vomiting, diarrhea and bleeding. Early diagnosis is done by detecting virus in blood with serological techniques and molecular tests. There is no cure for the disease and treatment is only supportive, through rehydration. Potential vaccines are undergoing human safety testing, but are not yet in clinical use.

Ebola quickly escalated from medical into a humanitarian, social, economic, and security crisis. Hospitals, schools, markets, businesses, tourism, traffic and borders were closed [6]. Ebola epidemic also has had a psychological impact, because fear and panic spreads much faster than the virus. The high fatality rate and lack of cure were the main reasons to provoke this fear. The context of the actual epidemic in West Africa is complex. Countries in this part of the world have gone through decades of civil war leading to destruction of their resources and health care systems, which are characterized with low numbers of health care workers and insufficient capacities in surveillance and information. Other weaknesses of these health care structures that had impact in the current outbreak are the lack of rapid response systems, small numbers of clinical laboratories, insufficient supplies of personal protective equipment (PPE), lack of water and electricity in health care facilities, low level of health education of population and inadequate involvement of communities in response to the epidemic [7].

Many hospitals were closed because of fears of the health care workers. Predominantly ambulatory services remained open. Guidelines of international organizations (WHO, CDC, MSF) very often remained solely on paper and were seldom executed in the field. Dissemination of disease in capital cities made contact investigation a great challenge. Other challenges in the field were waste management in health care facilities and coordination between national and international partners. Response from the international community did not parallel the exponential growth of the disease in the field. Nevertheless, it had a great impact in response to the epidemic. Community care centers were institutionalized in some countries looking for alternative ways to decrease the number of cases. After the outbreak all existing health care resources were dedicated to Ebola response, hitting health care services for other diseases affecting the population, such are chronic diseases, immunization and malaria (which causes 21% of deaths in children under the age of 5 in Liberia) [8].

The primary pillars to prevent EVD are: early diagnosis, isolation of patients, contact tracing and monitoring, safe burials, infection prevention and control and increased social mobilization. The implementation of all these components was challenged in the field. To address them affected countries must empower their national health care systems. They also need to establish proactive response systems for similar threats in the future.

The Ebola epidemic has initiated preparedness and response all over the world. Fortunately, the risk of transmission in European countries remains low. But, since nobody can predict where a patient with Ebola might go, each hospital and health care facility must be ready to and must evaluate the risk and procedures to isolate a patient with Ebola. Key points of preparedness are facility leadership, written and rehearsed standardized operating procedures, staff training and oversight of practices of PPE.

The current Ebola outbreak entered its second year. Although the disease curve is decreasing, it is not yet under control [9]. At the end of this outbreak we require debriefing to evaluate interventions, identify institutional gaps, and address economic and social consequences and plan for the rebuilding of health care systems.

Key lessons learnt during the Ebola outbreak: countries with weak health care systems can’t withstand the major outbreaks; preparedness to treat the first confirmed cases is a national emergency; all control measures must be coordinated together and community engagement is the great factor that contributes to the success of Ebola story.

*Authors have been part of Ebola response team in Liberia during 2014 as consultants of World Health Organization*.
